# Development of an unsupervised cycle contrastive unpaired translation network for MRI‐to‐CT synthesis

**DOI:** 10.1002/acm2.13775

**Published:** 2022-09-28

**Authors:** Jiangtao Wang, Bing Yan, Xinhong Wu, Xiao Jiang, Yang Zuo, Yidong Yang

**Affiliations:** ^1^ Department of Engineering and Applied Physics University of Science and Technology of China Hefei Anhui China; ^2^ Cancer Center, Sichuan Academy of Medical Sciences · Sichuan Provincial People's Hospital Chengdu Sichuan China; ^3^ Department of Radiation Oncology The First Affiliated Hospital of USTC Division of Life Sciences and Medicine University of Science and Technology of China Hefei Anhui China

**Keywords:** contrastive unpaired translation network, cycle‐consistent generative adversarial network, MRI‐only workflow, synthetic CT

## Abstract

**Purpose:**

The purpose of this work is to develop and evaluate a novel cycle‐contrastive unpaired translation network (cycleCUT) for synthetic computed tomography (sCT) generation from T1‐weighted magnetic resonance images (MRI).

**Methods:**

The cycleCUT proposed in this work integrated the contrastive learning module from contrastive unpaired translation network (CUT) into the cycle‐consistent generative adversarial network (cycleGAN) framework to effectively achieve unsupervised CT synthesis from MRI. The diagnostic MRI and radiotherapy planning CT images of 24 brain cancer patients were obtained and reshuffled to train the network. For comparison, the traditional cycleGAN and CUT were also implemented. The sCT images were then imported into a treatment planning system to verify their feasibility for radiotherapy planning. The mean absolute error (MAE), peak signal‐to‐noise ratio (PSNR), and structural similarity index (SSIM) between the sCT and the corresponding real CT images were calculated. Gamma analysis between sCT‐ and CT‐based dose distributions was also conducted.

**Results:**

Quantitative evaluation of an independent test set of six patients showed that the average MAE was 69.62 ± 5.68 Hounsfield Units (HU) for the proposed cycleCUT, significantly (*p*‐value < 0.05) lower than that for cycleGAN (77.02 ± 6.00 HU) and CUT (78.05 ± 8.29). The average PSNR was 28.73 ± 0.46 decibels (dB) for cycleCUT, significantly larger than that for cycleGAN (27.96 ± 0.49 dB) and CUT (27.95 ± 0.69 dB). The average SSIM for cycleCUT (0.918 ± 0.012) was also significantly higher than that for cycleGAN (0.906 ± 0.012) and CUT (0.903 ± 0.015). Regarding gamma analysis, cycleCUT achieved the highest passing rate (97.95 ± 1.24% at the 2%/2 mm criteria and 10% dose threshold) but was not significantly different from the others.

**Conclusion:**

The proposed cycleCUT could be effectively trained using unaligned image data, and could generate better sCT images than cycleGAN and CUT in terms of HU number accuracy and fine structural details.

## 1 INTRODUCTION

Malignant tumors have become a serious threat to human health.^1^, ^2^ Radiotherapy is one of the major treatment methods. Many patients can be cured by radiotherapy, and a considerable portion of patients can relieve symptoms and prolong survival by radiotherapy. Computed tomography (CT) is indispensable in current radiotherapy practice and is routinely used for radiation dose calculation and patient positioning correction. Poor soft tissue contrast is a major disadvantage of CT, leading to the imprecise delineation of treatment targets and organs at risk. Magnetic resonance imaging (MRI) has better soft tissue contrast and has been increasingly involved in the radiotherapy workflow. Recently on‐board MRI‐guided radiotherapy (MRgRT) has been implemented in the clinic. However, MRI lacks electron density information that is critical for accurate radiation dose calculation. One strategy to improve the MRgRT workflow is to generate synthetic CT (sCT) from which electron density information can be derived. Then, simulation CT may no longer be needed, and consequently, the image registration uncertainty between MRI and CT can also be eliminated. Currently, there is a geometrical uncertainty of approximately 2 mm in cranial CT/MRI image registration.^3^

The current methods for generating sCT from MRI can be divided into three categories: segmentation‐based methods,^4^, ^5^, ^6^ atlas‐based methods,^7^, ^8^, ^9^ and deep learning‐based methods.^10^, ^11^, ^12^ Although they differ greatly in algorithm, the general idea is to use the models developed based on preacquired MRI‐CT pairs to generate new sCT from incoming MRI. Segmentation‐based methods segment the MRI according to the preclassified tissue types and fill in the corresponding density value in each segmented tissue. These approaches are limited by the accuracy of the segmentation and the requirement to predetermine tissue types. Atlas‐based methods rely on elastic image registration. First, an MRI in the coregistered MRI‐CT atlas database is deformably registered to the new MRI, and then the same transformation is applied to the CT in the MRI‐CT pair to generate the sCT. These methods are limited by the accuracy of the elastic registration and lack robustness when large anatomical variations exist between the target and atlas MRI. Deep learning‐based methods are currently the method under intensive investigations. They can learn the complex and nonlinear mapping between MRI and CT images from a great number of MRI‐CT pairs. Due to this automatic learning feature, deep learning‐based methods are becoming increasingly popular in image synthesis tasks.^13^ In deep learning‐based methods, an sCT can be obtained within seconds using a well‐trained network.

The deep‐learning networks used in sCT generation can be mainly categorized into convolutional neural networks (CNNs),^14^, ^15^, ^16^ generative adversarial networks (GANs),^17^, ^18^, ^19^ and cycle‐consistent adversarial networks (cycleGANs).^20^, ^21^, ^22^ The complexity of these networks increases sequentially, and the former is the cornerstone of the latter. CNN evolved from multilayer perceptron (MLP). Due to its structural characteristics of local area connection, weight sharing, and downsampling, CNN performs well in the field of image processing. A major limitation of the CNN‐based methods is that they require strictly registered MRI‐CT pairs for the training task. GAN introduces an additional discriminator to distinguish the generated sCT from the real CT and adds an adversarial loss term in the loss function to generate more realistic sCT images. Unfortunately, GAN still requires decently aligned MRI‐CT pairs which are usually difficult to obtain. CycleGAN addresses this problem by introducing inverse mapping and cycle‐consistency loss. CycleGAN has attracted great interest because they enable unpaired MRI‐to‐CT transformation. In cycleGAN, the target appearance is enforced using an adversarial loss, while the image content is preserved using a cycle‐consistency loss. However, the cycle‐consistency loss assumes that the relationship between the two domains is a bijection, which is often too restrictive.^23^

To overcome the above limitations, Park et al.^23^ proposed a contrastive unpaired translation network (CUT) using an alternative but rather straightforward way of maintaining correspondence in the image content but not appearance by maximizing the mutual information between the corresponding input and output patches. It was successfully applied to horse‐to‐zebra, cat‐to‐dog, and cityscape related training tasks. In this work, we developed a novel cycle‐contrastive unpaired translation network (cycleCUT) by combining cycleGAN and CUT to improve the training performance using unpaired MRI and CT images. A compound loss function was also introduced in the cycleCUT to robustly predict more realistic sCT images. The proposed network should learn the voxel‐to‐voxel correspondence between MRI and CT images and meanwhile preserve the shape of anatomical structures. It is also expected that the generated sCT should have similar image contrast as the real CT.

## 2 MATERIALS AND METHODS

### 2.1 Image acquisition and preprocessing

Thirty brain cancer patients who received radiotherapy at the First Affiliated Hospital of USTC were included in this study. The data of each patient included routine planning CT and diagnostic MRI. The CT images were acquired on a GE scanner (Discovery CT590 RT, GE Healthcare Technologies, Milwaukee, Wisconsin, USA) with the following scanning parameters: 120 kV tube voltage, 416 mA tube current, 0.98 × 0.98 × 2.5 mm^3^ resolution, and 512 × 512 matrix size. The post‐gadolinium T1‐weighted MRI images were acquired with a brain volume imaging (BRAVO) sequence on a 1.5 T MRI scanner (Signa HDxt, GE Healthcare Technologies, Milwaukee, Wisconsin, USA). The scanning parameters were as follows: 7.4–8.4 ms repetition time, 2.4–3.1 ms echo time, 15° flip angle, and 0.47 × 0.47 × 0.47 mm^3^ resolution. The time interval between MRI and CT acquisition was less than 2 days. Image preprocessing was conducted with the following steps:

1. All MRI images were corrected by the N4ITK bias correction algorithm^24^ in MIM software to reduce the intensity inhomogeneity caused by biased magnetic fields. A histogram matching method^25^ was then used to standardize the scale of the MRI signal intensity.
2. The MRI images were rigidly registered to the corresponding CT images using MIM software. The aligned MRI and CT images were then resampled to a resolution of 1 × 1 × 1 mm^3^ and cropped to a size of 240 × 240.
3. A binary body mask excluding the couch, head mask, and other patient immobilization devices was obtained based on the CT images and propagated to the MRI images. The values outside the body mask were set to −1024 HU for CT images and 0 for MRI images.
4. Considering that the activation function used in the output layer of the generator in our model was “tanh,” all MRI and CT images were normalized to [−1, 1] according to their minimum and maximum intensity values, respectively.

### 2.2 Network model

#### 2.2.1 CycleGAN

**FIGURE 1:**
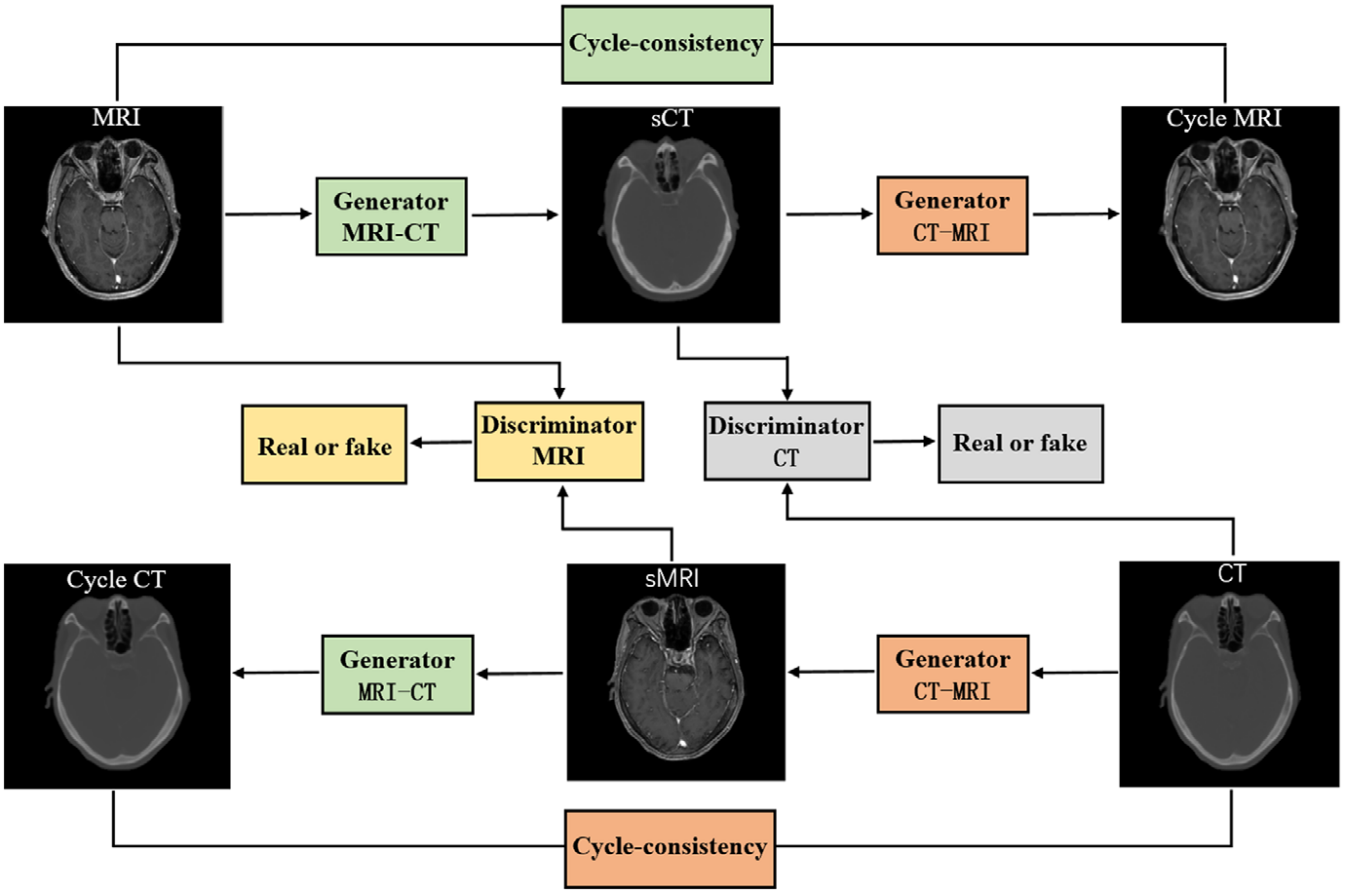
Schematic flowchart of cycleGAN. Two cycles are formed in cycleGAN. Two generators (i.e., $G_{MRI-CT}$ and $G_{CT-MRI}$) and two discriminators (i.e., $D_{CT}$ and $D_{MRI}$) are included. $G_{MRI-CT}$ is used to generate synthetic CT (sCT) from MRI and $G_{CT-MRI}$ is used to generate synthetic MRI (sMRI) from CT. $D_{CT}$ is used to distinguish CT from sCT and $D_{MRI}$ is used to distinguish MRI from sMRI. The cycle‐consistency concept assumes that the sCT image generated by a forward generator ($G_{MRI-CT}$) going through the opposite generator ($G_{CT-MRI}$) should result in a cycle MRI image that is equal to the real MRI image (and vice versa).

CycleGAN is a typical unsupervised learning network that can be trained using unpaired image data. This is due to the incorporation of an inverse transformation and the addition of a cycle‐consistency loss. Figure 1 shows the schematic flowchart of the cycleGAN network for MRI‐based sCT generation. It consists of two generators (i.e., $G_{MRI-CT}$ and $G_{CT-MRI}$) and two discriminators (i.e., $D_{CT}$ and $D_{MRI}$) and forms two cycles. $G_{MRI-CT}$ aims to generate sCT images that can fool the discriminator $D_{CT}$ into believing they are real CT images, while $D_{CT}$ aims to identify whether the images are real CT or sCT. The goals of $G_{CT-MRI}$ and $D_{MRI}$ are for the CT‐to‐MRI conversion and are the counterparts of $G_{MRI-CT}$ and $D_{CT}$. The concept of cycle consistency assumes that the sCT image generated by a forward generator ($G_{MRI-CT}$) going through the opposite generator ($G_{CT-MRI}$) should result in a cycle MRI image that is equal to the real MRI image (and vice versa). For more details, please refer to the original cycleGAN^26^ paper.

The loss function of cycleGAN includes the following terms: adversarial loss, cycle‐consistency loss, and identity mapping loss.^27^ Adversarial loss maps the distribution of the synthetic image to the distribution of the target image and is reflected in both generator and discriminator. $G_{MRI-CT}$ tries to minimize $L_{G\_A}$,

(1) $$L_{G\_A}=\frac{1}{m}\;\sum_{i\;=\;1}^{m}{\left(1-D_{CT}\left(G_{MRI-CT}\left(MRI_{i}\right)\right)\right)}^{2},$$

while $D_{CT}$ tries to minimize $L_{D\_A}$,

(2) $$\begin{array}{ccc} L_{D\_A} & = & \frac{1}{m}\\ & & \sum_{i=1}^{m}\frac{{\left(1-D_{CT}\left(CT_{i}\right)\right)}^{2}+{\left(D_{CT}\left(G_{MRI-CT}\left(MRI_{i}\right)\right)\right)}^{2}}{2}. \end{array}$$

Similarly, $G_{CT-MRI}$ tries to minimize $L_{G\_B}$, while $D_{MRI}$ tries to minimize $L_{D\_B}$:

(3) $$L_{G\_B}=\frac{1}{m}\;\sum_{i\;=\;1}^{m}{\left(1-D_{MRI}\left(G_{CT-MRI}\left(CT_{i}\right)\right)\right)}^{2},$$

(4) $$\begin{array}{ccc} L_{D\_B} & = & \frac{1}{m}\\ & & \;\;\sum_{i=1}^{m}\frac{{\left(1-D_{MRI}\left(MRI_{i}\right)\right)}^{2}+{\left(D_{MRI}\left(G_{CT-MRI}\left(CT_{i}\right)\right)\right)}^{2}}{2}. \end{array}$$

To enforce a one‐to‐one mapping, the cycle‐consistency losses for two cycles are incorporated in cycleGAN:

(5) $$L_{cycle\_MRI}=\frac{1}{m}\;\sum_{i\;=\;1}^{m}\left|G_{CT-MRI}\left(G_{MRI-CT}\left(MRI_{i}\right)\right)-MRI_{i}\right|,$$

(6) $$L_{cycle\_CT}=\frac{1}{m}\;\sum_{i\;=\;1}^{m}\left|G_{MRI-CT}\left(G_{CT-MRI}\left(CT_{i}\right)\right)-CT_{i}\right|.$$

If CT images are fed into $G_{MRI-CT}$, the results should also be CT, and vice versa. Thus, the identity mapping losses for MRI and CT are:

(7) $$L_{identity\_CT}=\frac{1}{m}\;\sum_{i\;=\;1}^{m}\left|\left(G_{MRI-CT}\left(CT_{i}\right)\right)-CT_{i}\right|,$$

(8) $$L_{identity\_MRI}=\frac{1}{m}\;\sum_{i\;=\;1}^{m}\left|\left(G_{CT-MRI}\left(MRI_{i}\right)\right)-MRI_{i}\right|.$$

Therefore, the full loss function for the two generators is,

(9) $$\begin{array}{ccc} L_{G\_cycleGAN} & = & L_{G\_A}\;+L_{G\_B}+\alpha \times \left(L_{cycle_{MRI}}+L_{cycle_{CT}}\right)\\ & & +\beta \times \left(L_{identity\_CT}+L_{identity\_MRI}\right), \end{array}$$

where α is 10 and β is 5, which are the common values set for cycleGAN.

The full loss function for the two discriminators is

(10) $$L_{D\_cycleGAN}=L_{D\_A}\;+L_{D\_B}.$$

#### 2.2.2 CUT

CUT can also be trained using unpaired image data. Unlike cycleGAN, CUT only needs to learn a mapping in one direction and consists of a generator and a discriminator. Adversarial loss is still used in CUT to encourage the generator to produce sCT images that are indistinguishable from real CT images. CycleGAN ensures the structural consistency of MRI and sCT through cycle consistency, while CUT uses a contrastive learning framework to maximize the mutual information between the two. The goal of contrastive learning is to associate two samples, a “query” and its “positive” patch, in contrast to other patches referred to as “negatives” within the image.

**FIGURE 2:**
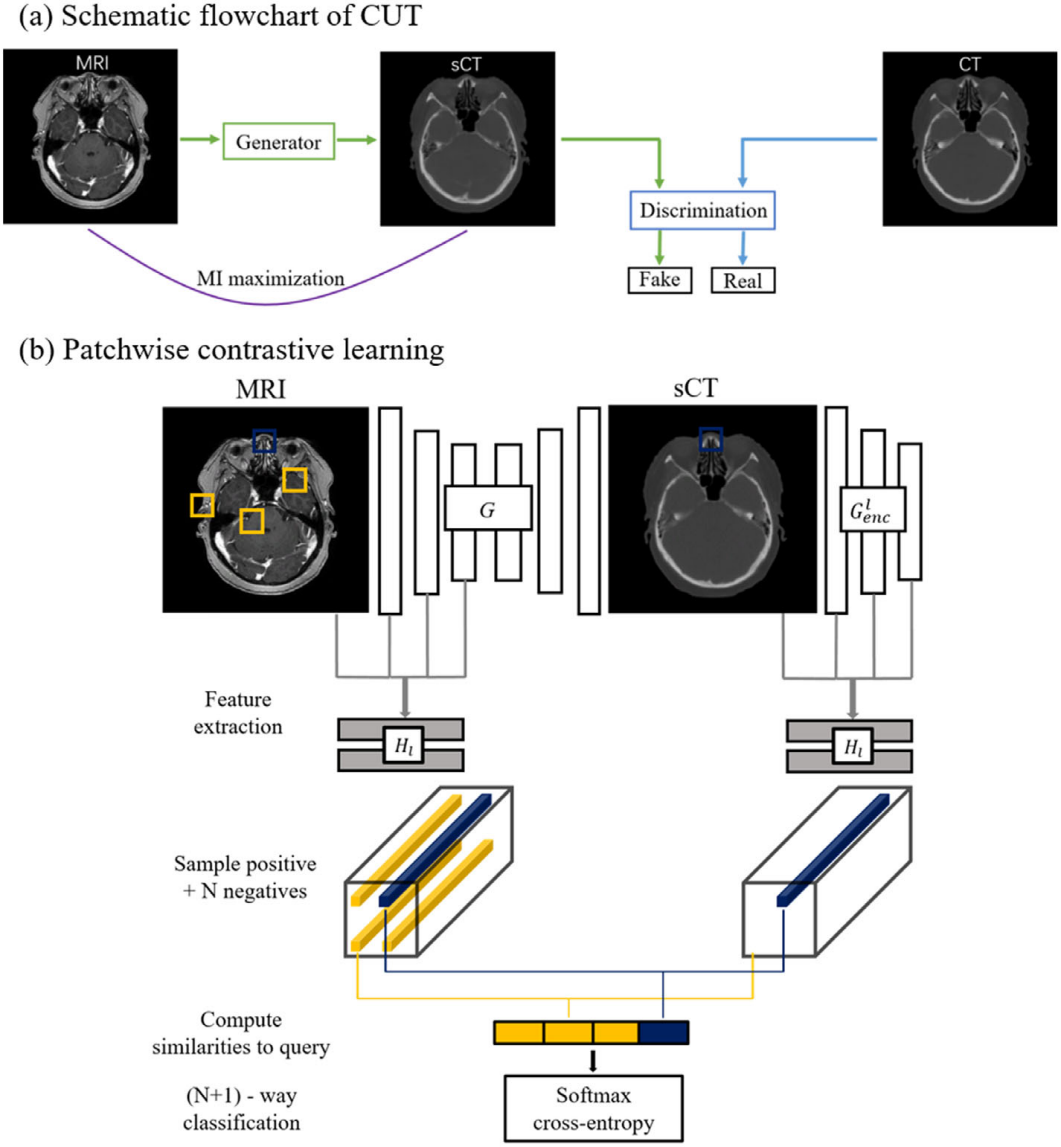
Schematic flowchart and patchwise contrastive learning frame of CUT. CUT only requires learning the mapping in one direction and consists of a generator and a discriminator. Patchwise contrastive learning encourages two corresponding patches to map to a similar point in the feature space, relative to other patches referred as negatives in the image.

The schematic flowchart and patchwise contrastive learning framework of CUT are shown in Figure 2. The generator is divided into two components: an encoder $G_{enc}$ and a decoder $G_{dec}$, which are applied sequentially to generate sCT (i.e., $sCT\;=\;G\left(MRI\right)\;=G_{dec}\;\left(G_{enc}\left(MRI\right)\right)$). The encoder $G_{enc}$ can be used not only to generate sCT by combining with $G_{dec}$ but also for available feature extraction. The *L*‐layer feature maps of interest are selected and then passed through a small two‐layer MLP network $H_{l}$, providing a stack of features: ${\left\{z_{l}\right\}}_{L}={\left\{H_{l}\left(G_{enc}^{l}\left(MRI\right)\right)\right\}}_{L}\;$ for MRI and ${\left\{{\hat{z}}_{l}\right\}}_{L}={\left\{H_{l}\left(G_{enc}^{l}\left(G(MRI)\right)\right)\right\}}_{L}\;$ for sCT, where $G_{enc}^{l}$ is the output of the *l*‐th chosen layer. PatchNCE loss $L_{PatchNCE}\left(G,H,MRI,sCT\right)$ is utilized to match corresponding MRI‐sCT patches at a specific location.

(11) $$\begin{array}{ccc} & & L_{PatchNCE}\;\left(G,H,MRI,sCT\right)=E_{\mathrm{MRI}∼P_{data\left(MRI\right)}}\;\\ & & \;\sum_{l\;=1}^{L}\sum_{s\;=1}^{S_{l}}f\left({\hat{z}}_{l}^{s},z_{l}^{s},z_{l}^{S\setminus s}\right), \end{array}$$

where $S_{l}$ denotes the number of spatial locations in each layer, $z_{l}^{s}$ represents the corresponding feature, $z_{l}^{S\setminus s}$ is the other features, and $f({\hat{z}}_{l}^{s},z_{l}^{s},z_{l}^{S\setminus s})$ is the cross‐entropy loss.

(12) $$\begin{array}{ccc} & & f({\hat{z}}_{l}^{s},z_{l}^{s},z_{l}^{S\setminus s})\\ & & =-\log\left[\frac{\exp\left({\hat{z}}_{l}^{s}\cdot z_{l}^{s}/\tau \right)}{\exp\left({\hat{z}}_{l}^{s}\cdot z_{l}^{s}/\tau \right)+\sum_{n\;=\;1}^{S_{l}-1}\exp\left({\hat{z}}_{l}^{s}\cdot {\left(z_{l}^{S\setminus s}\right)}_{n}/\tau \right)}\right] \end{array}$$

where τ is a scaling factor with a value of 0.07.

The identity loss $L_{PatchNCE}\left(G,H,CT,sCT\right)$ in the CT domain is used to prevent the generator from making unnecessary changes and to increase the training stability. Therefore, the full loss function for the generator of the CUT is

(13) $$\begin{array}{ccc} L_{G\_CUT} & = & L_{G\_A}\;+\lambda_{1}L_{PatchNCE}\left(G,H,MRI,sCT\right)\\ & & +\;\lambda_{2}L_{PatchNCE}\left(G,H,CT,sCT\right), \end{array}$$

where λ_1_ and λ_2_ are equal to 1. The full loss function for the discriminator is

(14) $$L_{D\_CUT}=L_{D\_A}\;.$$

#### 2.2.3 CycleCUT

**FIGURE 3:**
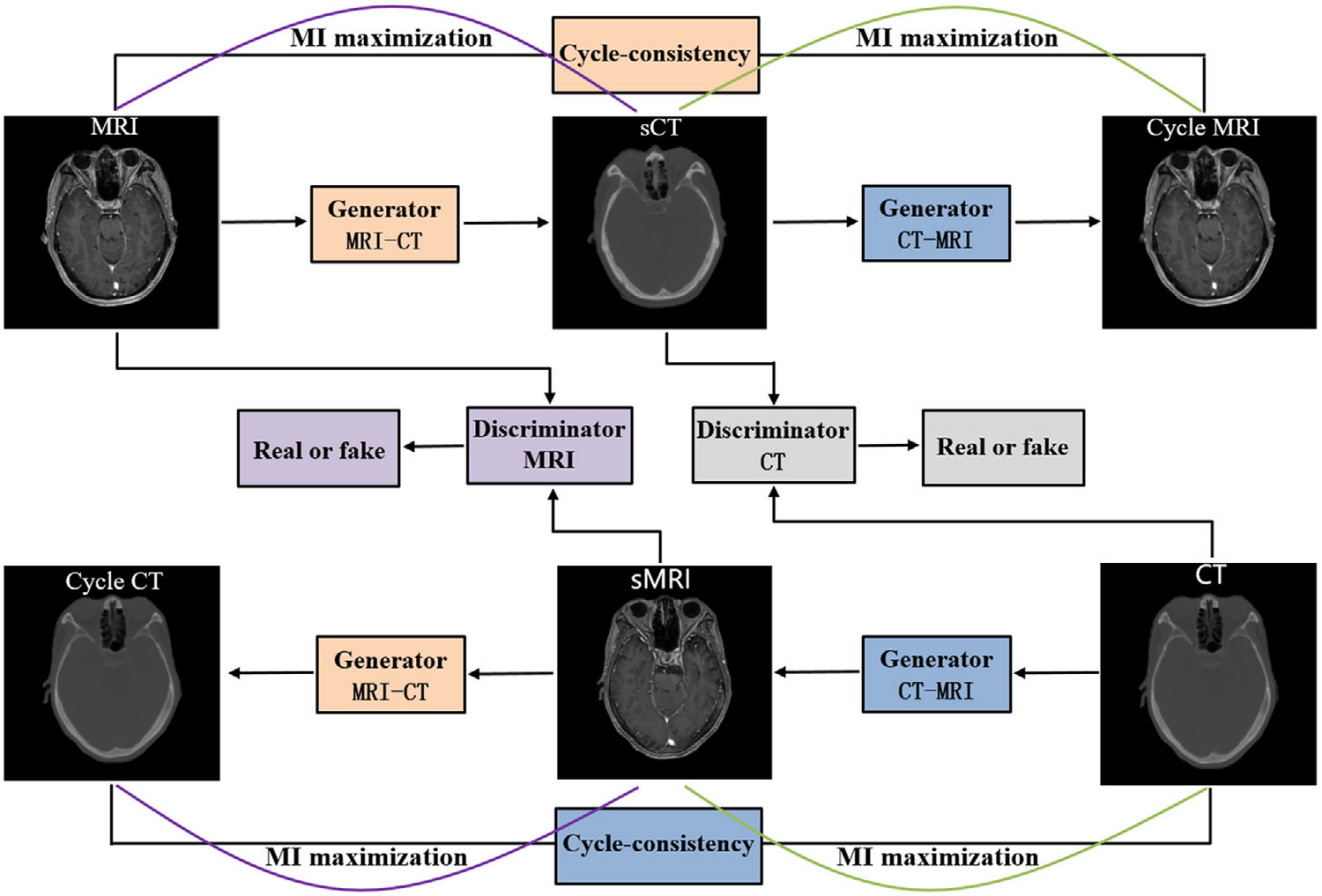
Schematic flowchart of cycleCUT. The cycleCUT consists of two generators, two discriminators, and two contrastive learning frameworks used to maximize the mutual information between the input and output of the two generators. The structural consistency between MRI and the corresponding sCT is guaranteed by the cycle‐consistency loss and patchwise contrastive learning simultaneously.

In this work, we developed a hybrid deep‐learning model combining CUT and cycleGAN networks. Specifically, we introduced the contrastive learning module of CUT into the cycleGAN framework and created a new network named the “cycle‐contrastive unpaired translation network” (cycleCUT). Figure 3 shows the schematic flowchart of the cycleCUT. Similar to cycleGAN, the cycleCUT consists of two generators and two discriminators. There are two contrastive learning frameworks to maximize the mutual information between the input and output of the two generators. The generator containing an encoder and a decoder and the discriminator consisting of three downsampling convolutional layers followed by a sigmoid layer in CUT were used for both cycleCUT and cycleGAN.

In the cycleCUT, the cycle‐consistency loss and patchwise contrastive learning are used simultaneously to ensure the structural consistency between MRI and the corresponding sCT. Thus, it can effectively distinguish the structure boundaries with significant HU variations and maintain the sharpness of the sCT.

The final loss function for the two generators of the cycleCUT is

(15) $$\begin{array}{c} \begin{array}{c} \;L_{G_{cycleCUT}}=L_{G_{A}}\;+L_{G_{B}}+\alpha \times \left(L_{cycle_{MRI}}+L_{cycle_{CT}}\right)\\ \;+\beta \times \left(L_{identity_{CT}}+L_{identity_{MRI}}\right)\\ \;+\eta_{1}L_{PatchNCE}\left(G_{MRI-CT},H_{MRI-CT},MRI,sCT\right)\\ \;+\eta_{2}L_{PatchNCE}\left(G_{MRI-CT},H_{MRI-CT},sMRI,cycle\_CT\right)\\ \;+\eta_{3}L_{PatchNCE}\left(G_{CT-MRI},H_{CT-MRI},CT,sMRI\right)\\ \;+\eta_{4}L_{PatchNCE}\left(G_{CT-MRI},H_{CT-MRI},sCT,cycle\_MRI\right) \end{array}, \end{array}$$

where α is 10, β is 5, and $\eta_{1}-\eta_{4}$ are equal to 1. The $\eta_{1}-\eta_{4}$ values were empirically set so that all the losses were roughly in the same order.

The loss function for the two discriminators of the cycleCUT is

(16) $$L_{D\_cycleCUT}=L_{D\_A}\;+L_{D\_B}.$$

### 2.3 Implementation details

**FIGURE 4:**
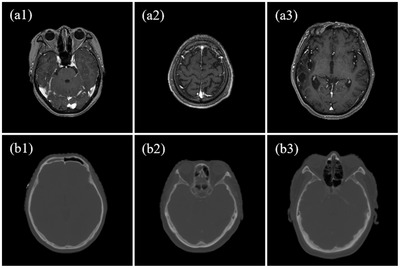
Unpaired training data. (a1–a3) are randomly selected MRI images in the training set, and (b1–b3) are the corresponding CT images. We trained all models in this work using MRI and CT images of different patients at different anatomical locations.

We divided the 30 patients included in this work into two groups: 24 for training and 6 for testing. To increase the number of training samples, each image was padded to 286 × 286 pixels and then randomly cropped to subimages of 256 × 256 pixels during training. Five percent rotation and random horizontal flip were also used for data augmentation. We trained all models with unpaired data for fair comparison among the three networks, that is, MRI and CT images of different patients at different anatomical locations were fed into the network simultaneously (Figure 4).

All models mentioned in this work were implemented in PyTorch with the Adam optimizer using the same training hyperparameters and strategies, and they were trained and tested on an NVIDIA GeForce RTX 3090 GPU (24G) with a batch size of 1. All models were trained for 200 epochs, with a fixed learning rate of 0.0002 for the first 100 epochs and a varied one linearly decaying to zero for the last 100 epochs.

### 2.4 Evaluation strategy

To evaluate the accuracy of sCT, three commonly used metrics were employed: mean absolute error (MAE), peak signal‐to‐noise ratio (PSNR), and structural similarity index (SSIM). They are calculated as follows:

(17) $$MAE\;=\frac{1}{N}\;\sum_{i\;=\;1}^{N}\left|CT_{i}-sCT_{i}\right|,$$

(18) $$PSNR\;=\;10log_{10}(\frac{Q^{2}}{CT-sCT_{2}^{2}/N}$$

(19) $$SSIM\;=\frac{\left(2\mu_{CT}\mu_{sCT}+C_{1}\right)\left(2\delta_{CT\cdot sCT}+C_{2}\right)}{\left(\mu_{CT}^{2}+\mu_{sCT}^{2}+C_{1}\right)\left(\delta_{CT}^{2}+\delta_{sCT}^{2}+C_{2}\right)}\;,$$

where *N* represents the number of voxels in the region of interest (ROI); *Q* is the maximum HU value of the two images; $C_{1}={\left(0.01Q\right)}^{2}\;$ and $C_{2}={\left(0.03Q\right)}^{2}\;$; $\mu_{CT}$ and $\mu_{sCT}$ are the average values of CT and sCT, respectively; $\delta_{CT}$ and $\delta_{sCT}$ are the standard deviations of CT and sCT, respectively; and $\delta_{CT\cdot sCT}$ is the covariance matrix between CT and sCT.

In addition, the global 3‐dimensional gamma passing rates with different criteria (2%/2 mm and 3%/3 mm) and different dose thresholds (10%, 30%, 50%, 70%, and 90% of the prescription dose) were also used to conduct dosimetry comparison. For each patient in the test set, an intensity‐modulated radiotherapy plan with 7 beams (30°, 90°, 140°, 180°, 220°, 275°, and 330°) of 6 MV photons was designed based on the real CT images using the Pinnacle treatment planning system (TPS). The corresponding sCT images were then imported into the TPS to recalculate the dose distribution by keeping all planning parameters unchanged. The prescription dose was 50 Gy, and all doses were calculated with a resolution of 1 × 1 × 1 mm^3^.

## 3 RESULTS

### 3.1 Image comparison

**FIGURE 5:**
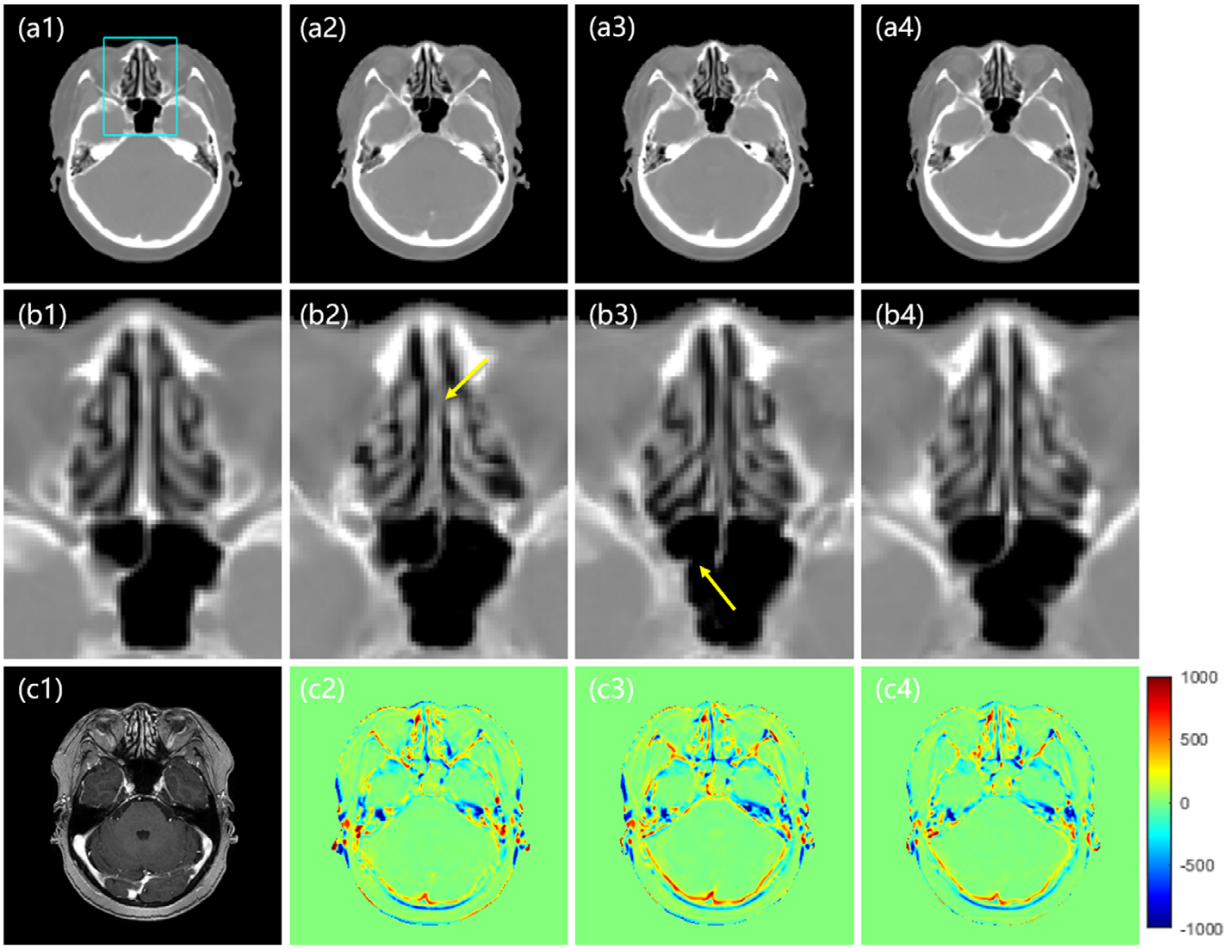
The sCT images in the axial plane. The first row shows the real CT (a1) and the sCT images generated by the cycleGAN method (a2), the CUT method (a3), and the proposed cycleCUT method (a4). Panels (b1–b4) highlight ROI outlined by the rectangle shown in (a1). The corresponding MRI is shown in (c1). (c2–c4) show the error images for each sCT, with the planning CT taken as the ground truth. Yellow arrows indicate the site of misclassification. The display window is [−160 240] for all CT images.

**FIGURE 6:**
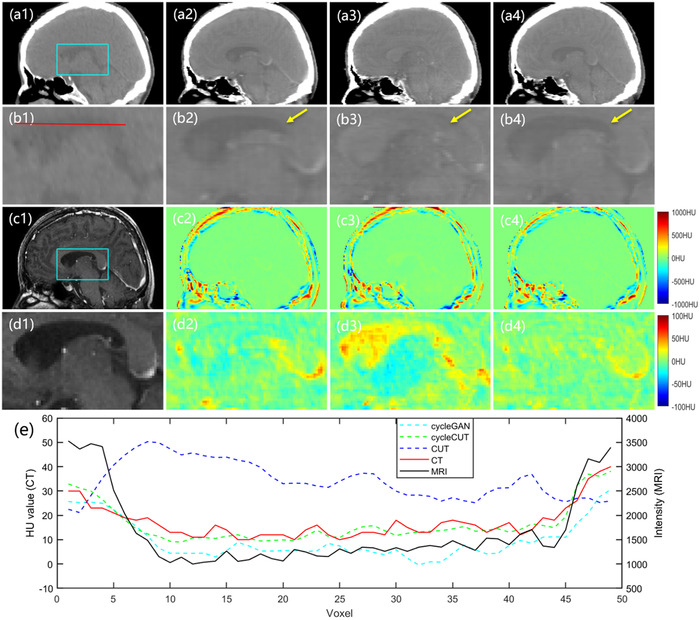
The sCT images in the sagittal plane. The first row shows the real CT (a1) and the sCT images produced by the cycleGAN method (a2), the CUT method (a3), and the proposed cycleCUT method (a4). Panels (b1–b4) highlight ROI outlined by the rectangle shown in (a1). The corresponding MRI and enlarged ROI are shown in (c1) and (d1), respectively. (c2–c4) show the error images for each sCT, with the planning CT taken as the ground truth. The enlarged ROI images are shown in (d2–d4). The profiles along the red line shown in (b1) are shown in (e). The display window is [−160 240] for all CT images.

To qualitatively evaluate the three different sCT generation methods, the axial, sagittal, and coronal views of an exemplary patient are shown in Figure 5, 6, 7. These slices represent some of the most challenging parts of the brain in sCT generation. Although they were trained using unpaired data, as shown in Figure 4, the networks were still able to convert MRI images to CT images while maintaining anatomical accuracy. Furthermore, the generated sCT images show image contrast similar to the real CT among various tissue types.

**FIGURE 7:**
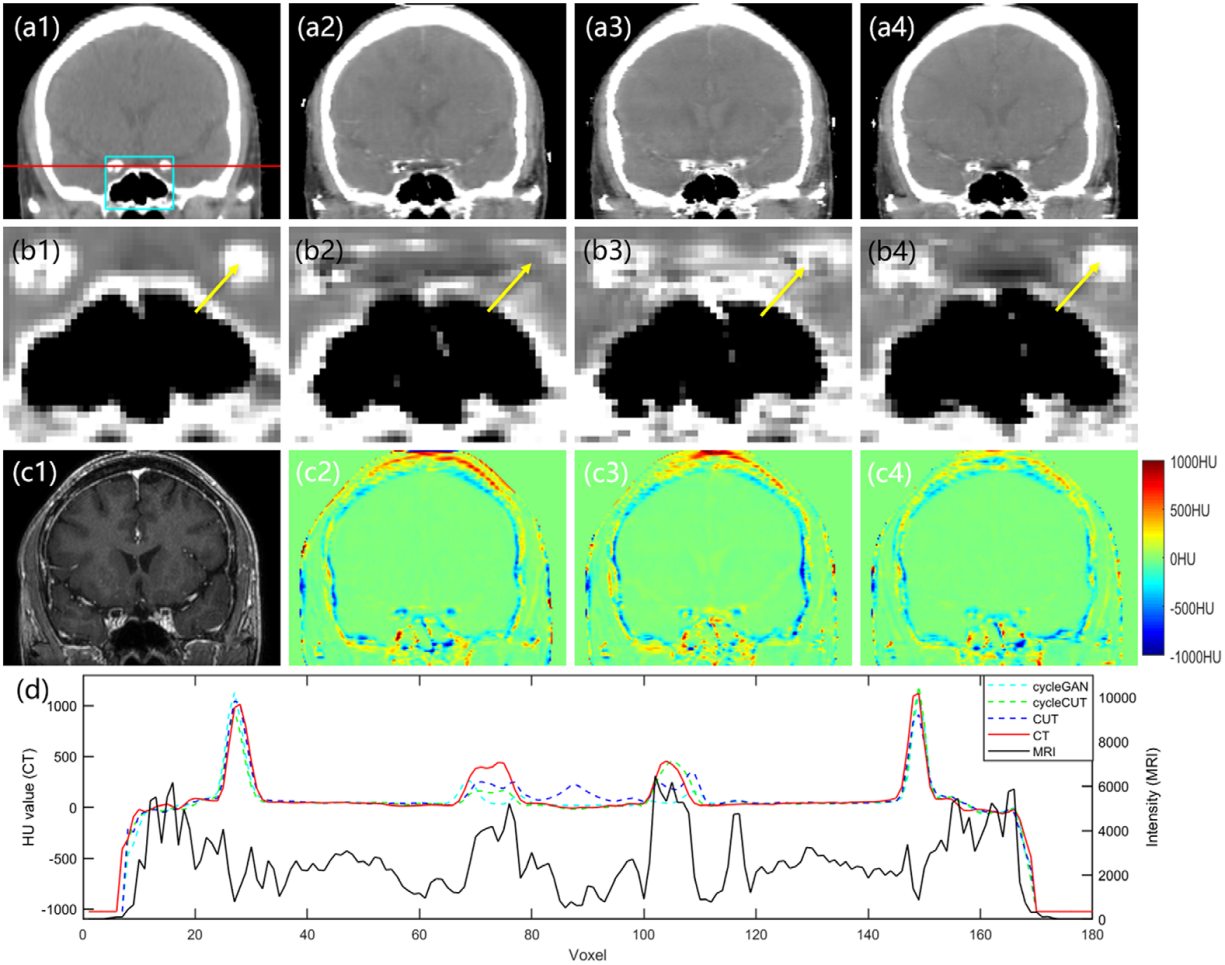
The sCT images in the coronal plane. The first row shows the real CT (a1) and the sCT images obtained by the cycleGAN method (a2), the CUT method (a3), and the proposed cycleCUT method (a4). Panels (b1–b4) highlight the ROI outlined by the rectangle shown in (a1). The corresponding MRI is shown in (c1). (c2–c4) show the error images for each sCT, with the planning CT taken as the ground truth. The profiles along the red line shown in (a1) are shown in (d). The display window is [−160 240] for all CT images.

Figure 5 shows the axial views and the corresponding error images in sCT generation. Panel (a1) shows a real CT in the axial view, (c1) is the corresponding MRI, and (a2–a4) show sCT images generated by cycleGAN, CUT, and the proposed cycleCUT, respectively. Panels (b1–b4) are the enlarged images corresponding to (a1–a4) within the ROI outlined by the rectangle shown in (a1). Panels (c2–c4) are the corresponding error images of (a2–a4), with the planning CT taken as the ground truth. The ROI in (a1) is selected at a location with large anatomical variations and probably represents the most challenging region in sCT generation. As marked by the yellow arrows, there are some misclassified voxels in the sCT images generated by cycleGAN (b2) and CUT (b3). In contrast, the sCT (b4) produced by the proposed method has superior CT HU accuracy and better preservation of structural details.

Figure 6 shows the sagittal views. Panel (a1) shows a real CT in the sagittal view, (c1) is the corresponding MRI, and (a2–a4) are sCT images produced by cycleGAN, CUT, and the proposed cycleCUT, respectively. Panels (b1–b4) are the enlarged images corresponding to (a1–a4) within the ROI outlined by the rectangle shown in (a1). Panels (c2–c4) are the corresponding error images of (a2–a4), with the planning CT taken as the ground truth. Panels (d1–d4) show the enlarged ROI. The profiles on (b1–b4) and (d1) along the red line in (b1) are shown in (e). As marked by the yellow arrows, the ventricle in the sCT generated by the cycleCUT (b4) has the closest appearance to that in the real CT. The error images (d2–d4) and the line profiles in (e) also demonstrate that the HU distribution in the sCT generated by the cycleCUT is the closest to the real CT. These results indicate that the proposed method outperforms the cycleGAN and CUT, both in terms of definitive tissue boundaries and accurate HU values.

Figure 7 shows the coronal views. The first row shows the real CT (a1) and the sCT images obtained by cycleGAN (a2), CUT (a3), and the proposed method (a4). The second row highlights the ROI, and the third row shows the corresponding MRI and error images. Figure 7 demonstrates excellent agreement with the ground truth for the sCT generated by the proposed method, especially at the location marked by the yellow arrow. Furthermore, the profiles along the red line in (a1–a4) and (c1) are shown in (d). These results suggest the sCT generated by the cycleCUT has a HU distribution closest to the real CT.

**FIGURE 8:**
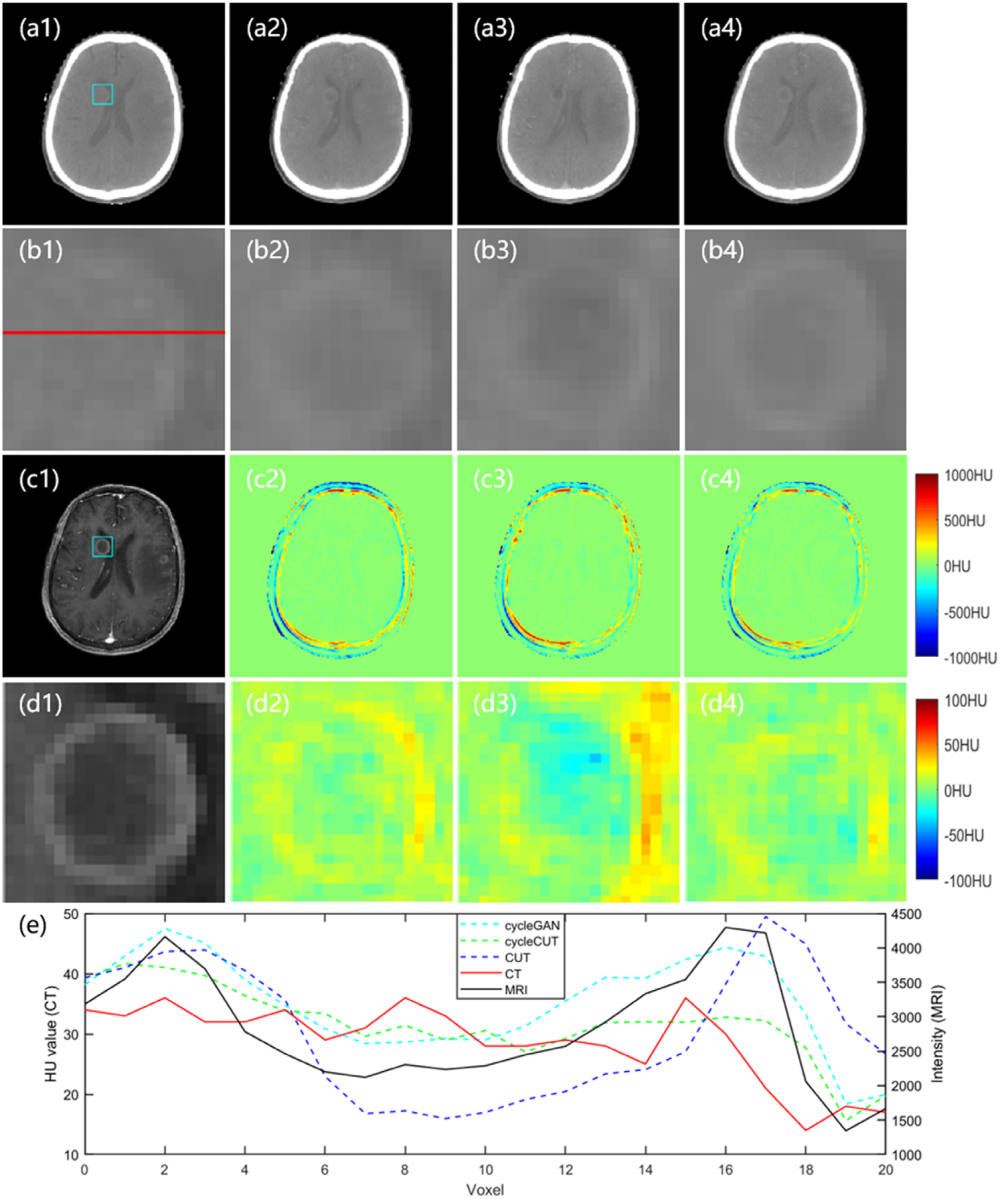
The sCT images with a tumor. The first row shows the real CT (a1) and the sCT images generated by the cycleGAN method (a2), the CUT method (a3), and the proposed cycleCUT method (a4). Panels (b1–b4) highlight the tumor region in (a1–a4) for the rectangle ROI shown in (a1). The corresponding MRI and highlighted ROI are shown in (c1) and (d1). Panels (c2–c4) show the error images for each sCT, with the planning CT taken as the ground truth, and the ROI images are shown in (d2–d4). The profiles along the red line shown in (b1) are shown in (e). The display window is [−160 240] HU for all CT images.

Figure 8 shows images with a tumor. The first row shows the real CT (a1) and the sCT images produced by cycleGAN (a2), CUT (a3), and the proposed method (a4). The second row shows the highlighted tumor region, and the third row shows the corresponding MRI and error images. The fourth row highlights the rectangle ROI corresponding to (c1–c4). The fifth row shows the profiles along the red line in (b1). The image contrast between the tumor and the surrounding soft tissues is obvious in MRI but is reduced in CT. As indicated by the error images (d2–d4) and the line profiles in (e), the HU values across the tumor region in the sCT produced by the proposed cycleCUT method are closest to those in the real CT.

### 3.2 Quantitative evaluation

The quantitative analysis results are summarized in Table 1, where MAE, PSNR, and SSIM between sCT and real CT are calculated. Among all the methods mentioned in this work, the proposed cycleCUT gives the smallest MAE of 69.62 HU, the largest PSNR of 28.73 dB, and the largest SSIM of 0.918, indicating that the proposed method outperforms the other methods. A two‐tailed paired *t*‐test was also conducted to verify whether the improvement was significant. The results in Table 1 show that there is a statistically significant improvement between cycleCUT and the other methods (*p*‐value < 0.05), while there is no statistically significant difference between CUT and cycleGAN (*p*‐value > 0.05)

**TABLE 1:** Comparison of different methods on sCT images

	MAE (HU)	PSNR (dB)	SSIM
cycleGAN	77.02 ± 6.00	27.96 ± 0.49	0.906 ± 0.012
CUT	78.05 ± 8.29	27.95 ± 0.69	0.903 ± 0.015
cycleCUT	69.62 ± 5.68	28.73 ± 0.46	0.918 ± 0.012
*p*‐Value of cycleGAN vs. CUT	0.486	0.938	0.424
*p*‐Value of cycleCUT vs. CUT	0.004	0.004	0.009
*p*‐Value of cycleCUT vs. cycleGAN	<0.001	<0.001	<0.001

### 3.3 Dosimetric evaluation

We also compared the dose calculation accuracy on sCT images obtained by different methods. The gamma passing rates for the 2%/2 mm and 3%/3 mm criteria with 10%, 30%, 50%, 70%, and 90% dose thresholds are listed in Table 2. For all methods, the gamma passing rates are greater than 97%. It can be seen that the dose calculation accuracy on sCT from the cycleCUT is slightly better than the others. However, there is no statistically significant difference between different methods.

**TABLE 2:** Comparison of gamma passing rates between different methods

	**2%/2** **mm**			**3%/3** **mm**		
**Dose threshold**	**CUT (%)**	**cycleGAN (%)**	**cycleCUT (%)**	**CUT (%)**	**cycleGAN (%)**	**cycleCUT (%)**
10%	97.59 ± 2.56	97.22 ± 2.74	97.95 ± 1.24	99.04 ± 1.02	98.68 ± 1.05	99.02 ± 0.60
30%	97.93 ± 2.07	97.86 ± 2.63	98.08 ± 1.55	99.14 ± 1.27	98.88 ± 1.47	99.22 ± 1.01
50%	97.25 ± 2.91	97.11 ± 3.79	97.34 ± 2.32	98.73 ± 2.22	98.28 ± 2.36	98.89 ± 1.49
70%	97.87 ± 2.66	97.34 ± 3.54	98.00 ± 2.10	99.07 ± 2.00	98.61 ± 1.92	99.35 ± 1.01
90%	99.62 ± 0.86	99.15 ± 1.18	99.80 ± 0.43	99.98 ± 0.04	99.99 ± 0.01	100.00 ± 0.00

## 4 DISCUSSION

Synthetic CT generation, from which electron density information can be derived, is critical in MRI‐only radiotherapy workflow. Most of the existing methods used to generate sCT from MRI require a training set of paired MRI and CT images. Given the scarcity of paired MRI and CT data, we developed a novel cycleCUT network by combining two typical unsupervised deep learning networks, cycleGAN and CUT. Correspondingly, a hybrid loss function was also introduced in the cycleCUT to robustly predict more realistic sCT images. Since the activation function used in the output layer of the generator in our model is “tanh” and the output range of the tanh function is [−1 1], both CT and MRI images are normalized to [−1 1] according to their respective maximum and minimum intensity values. For CT images, the intensity values have HU units, so all CT images have similar intensity ranges. For MRI images, a histogram matching method is used to standardize the scale of the MRI signal intensity before normalizing to [−1 1]. After standardization, the intensity values of all MRI images are in similar ranges. Therefore, normalizing to [−1, 1] does not cause any obvious shifting of data from one scan to another.

The results showed that the cycleCUT could be effectively trained using unpaired data, which would relax many restrictions on the data for current deep learning‐based sCT generation methods. The images for different medical purposes could all be collected in a large dataset and utilized to train the cycleCUT. The qualitative analysis demonstrated that CT images produced by the proposed cycleCUT method appeared more realistic and contained fewer artifacts than those produced by the cycleGAN and CUT methods. The quantitative evaluation showed that cycleCUT achieved higher accuracy in predicting the HU values than cycleGAN and CUT. However, the dosimetric improvement was minimal. This is probably due to the insensitivity of the MV photon dose calculation to small CT value variation. It is worth mentioning that proton radiotherapy may benefit more from the improved sCT image quality because the proton stopping power is more sensitive to HU value changes. In addition, better image quality would also generate better DRR images to assist patient setup.

As shown in the error images in Figure 5, 6, 7, all sCT images have small HU errors in the soft tissue but relatively large HU errors at the tissue interfaces. The larger error at the interface is partially caused by the nonperfect registration between the MRI and CT images. Because the MRI and CT images were not acquired at the same time, the anatomical structures of sCT which are derived from the MRI cannot be completely registered to those in CT, even if the MRI‐to‐sCT conversion is perfect. Therefore, the prediction error of sCT inevitably contains the registration error during the result evaluation and is more notable at the tissue interfaces. In addition, the blurred boundary at the bone–air interface in T1‐weighted MRI may also result in large HU errors in sCT generation because bone has a similar appearance to air in T1‐weighted MRI. Its impact on the radiation dose calculation, especially near the bone–air boundary, warrants further evaluation. Qi et al.^18^ showed that using multiple MRI sequences as model input could obtain better results than using one single MRI sequence. The ultrashort echo time (UTE) sequence can provide better bone signals, so adding UTE sequence MRI to the input can help distinguish the bone–air boundary in sCT.

Due to the limitation of GPU memory and small dataset, the three models in this study were all trained using 2D images, which might result in poor continuity for the sCT along the image thickness dimension. As shown in Figures 6 and 7, although the cycleCUT achieves better results than cycleGAN and CUT, the sCT images still have blurs and noise artifacts in the sagittal and coronal planes. In the future, a 3D model using multiple MRI sequences as input and trained with a larger dataset may be developed to improve the network performance. In addition, MRI images from different scanners may have different image quality. It is unclear how the method will be affected if the training dataset is from one scanner and the test dataset is from another. Future studies will incorporate patient images acquired from different scanners to test the scope of the clinical application of the proposed method.

## 5 CONCLUSION

In this study, we proposed a novel deep learning‐based method that integrated CUT and cycleGAN networks to generate sCT images from MRI. The proposed network could be effectively trained with unpaired MRI‐CT data and outperformed both cycleGAN and CUT in terms of both structural details and HU accuracy. This method could be applied in radiotherapy for sCT generation to accelerate the MRI‐only treatment workflow.

## AUTHOR CONTRIBUTIONS

Jiangtao Wang: Study design, data analysis, and manuscript drafting; Bing Yan: data collection and manuscript revision; Xinhong Wu: study design and manuscript revision; Xiao Jiang: data analysis and manuscript revision; Yang Zuo: data analysis and manuscript revision; Yidong Yang: study guidance, manuscript revision, and financial support.

## CONFLICT OF INTEREST

The authors have no conflict of interest to disclose.
